# Comparison on clinical efficacy and adverse reactions between TPICC and ultrasound-guided PICC for advanced tumors: A retrospective study

**DOI:** 10.1097/MD.0000000000038130

**Published:** 2024-10-18

**Authors:** Yingshan Zheng, Xia Xiang, Lixiang Li, Li Zhang, Shaoli He

**Affiliations:** a Intravenous Catheter Care Center, The First People’s Hospital of Foshan, Foshan, Guangdong, China; b Nursing Ministry, The First People’s Hospital of Foshan, Foshan, Guangdong, China; c Oncology Department, The First People’s Hospital of Foshan, Foshan, Guangdong, China.

**Keywords:** advanced tumors, adverse reactions, clinical efficacy, TPICC (Tunnel PICC), ultrasound guidance

## Abstract

Comparing the differences in efficacy and adverse reactions on the application between tunnel peripherally inserted central catheter (TPICC) and ultrasound-guided peripherally inserted central catheter (PICC) in patients with advanced tumors. A retrospective investigation was conducted to collect treatment data. We randomly selected 200 patients with advanced tumors who were admitted to our hospital from January 2020 to January 2022 as the research subjects. The observation group consisted of 100 cases of tunnel PICC catheterization, while the control group consisted of 100 cases of PICC catheterization. We observed and compared the catheterization time, PICC puncture success rate, intraoperative blood loss, pain degree, comfort level, and postoperative complication rate between the 2 groups. Compared with the control group, there was no significant difference at the distribution of PICC indwelling time in the observation group, and the difference was not statistically significant (*P* > .05). The success rate on PICC puncture in the observation group was higher than that on the control group (*P* < .05). Intraoperative bleeding volume and numerical rating scale (NRS) of the observation group were lower than those of the control group (*P < *.05). At 1 month postoperatively, comfort ratings in the observation group and the control group were lower than those of their same groups at 1 week postoperatively (*P < *.05); At 1 week and 1 month postoperatively, comfort rating in the observation group were lower than that in the control group (*P* < .05). The incidence of postoperative complication in the observation group was lower than that in the control group (*P* < .05). TPICC improves the success rate on puncture and the post-catheterization comfort, it reduces NRS and the risks on complications, which owns high safety.

## 1. Introduction

Peripherally inserted central catheter (PICC), is a peripheral vein puncture, catheter tip enters into central vein. Drugs are directly infused in central vein for avoiding the damage on blood vessels caused by long-term infusion or the infusions from hypertonic and irritating drugs in order to alleviate the pain caused by repeated puncture and improve the quality of life among patients,^[1]^ which is now widely used in the chemotherapy of tumors or other diseases,^[2,3]^ the parenteral nutritional pathway for adults postoperatively,^[4]^ and the creation of nutritional pathway for preterm infants,^[5]^ and so on. Although there are many advantages of PICC, such as avoiding the stimulation of blood vessels caused by drugs for patients who receiving oncology chemotherapy^[6]^ and reducing the incidence of drug extravasation and the rate of unplanned extubation, etc.^[7]^ However, some studies have confirmed that the incidence of its related complications is 16.35%.^[8]^ During PICC intubation, due to improper operation and other reasons, patients may experience catheter separation and secondary dislodgement, resulting in unplanned extubation. The incidence of PICC-related complications not only increases the physical burden of patients, but also increases the cost of treatment. It has been found in studies that the incidence of these problems is related to various factors such as the placement position of catheter, sealing method and catheter material, etc.^[8–10]^ Therefore, it is extremely important to improve puncture methods for effectively reducing complications and upgrading puncture effects.

TPICC is done by creating a subcutaneous tunnel. It makes the vascular puncture point at a certain distance from the catheter exit and shifts the catheter exit to a more suitable and advantageous position.^[11,12]^ TPICC solves the difficult problems of intravenous catheterization for many patients clinically, such as the small diameter of the vein or the presence of branching and the variations in the blood vessels, scarring lesions on the skin, extreme emaciation, skin laxity, a higher risk of infections, poor coagulation function or hypo-proteinemia, etc. TPICC has advantageous on protecting the patient’s peripheral vasculature, reducing the times of repeated puncture, alleviating the patient’s pain, diminishing the associated infections, decreasing the complication incidence and improving the comfort of intubation. Studies have shown that the central-tunnel catheters are more stable and may have a lower infection rate than non-tunnel catheters.^[13]^ In this study, we compared and observed patients with advanced tumors who were applied with TPICC or ultrasound-guided PICC respectively to understand the differences between these 2 PICCs and investigate the differences in the efficacy and adverse effects of TPICC and ultrasound-guided PICC in patients with advanced tumors.

## 2. Information and methodology

### 2.1. Study subjects

In this retrospective study, this study was approved by The Ethics Committee of The First People’s Hospital of Foshan. We randomly selected 200 patients with advanced tumors who were admitted to our hospital from January 2020 to January 2022 as research subjects. The patients were divided into 2 groups: an observation group (n = 100) and a control group (n = 100), based on whether they received B-ultrasound guidance. Inclusion criteria: Following with the doctor’s instructions for the catheterization before the initial chemotherapy; Age ≥ 18 year old; Diagnosed with malignant tumor by pathological examination, and the pathological stage is IV; The patients are conscious and can cooperate normally; The skin at the puncture site is intact, and the vein in the upper arm is in line with the conditions of puncture. Exclusion criteria: Absolute contraindications on PICC^[14]^; Coagulation disorders or platelet count < 20 × 10^9^/L; Death of the patient during the study period, or the subjective factor such as the abandonment of treatment leading to unplanned extubation. All patients and their families signed the informed consent. And all processes of this study were in accordance with the *Declaration of Helsinki*.

### 2.2. Methods

This study utilized a retrospective investigation to collect treatment data. The observation group comprised 100 patients who underwent PICC puncture by naked eye or were unable to undergo blind puncture. Tunnel PICC catheterization was performed. The average age of the observation group was 43.64 ± 5.57 years. The control group consisted of 100 patients with advanced tumors who underwent B-ultrasound-guided placement, with an average age of (45.05.4–56.89) years. There were no significant differences between the 2 groups in terms of gender, age, and disease type, making them comparable.

### 2.3. Evaluation indicators

#### 2.3.1. Indwelling Time of PICC

The time started from the skin disinfection and ended with the dressing fixation.

#### 2.3.2. Success rate on PICC puncture

The norms of successful catheterization: The successful puncture and the exact location of catheterization, that is, the catheter tip reaching within the lower third of superior vena cava or nearing the binding site of right atrium, and the tip of femoral venous catheter should rest in the inferior vena cava at the level above the diaphragm.

#### 2.3.3. Total bleeding volume in catheterization

Sterile gauze which were used up before and after catheterization were placed on a precise electronic scale and the difference in the numerical values according to the weigh was the amount of bleeding.

#### 2.3.4. Numerical rating scale

Numerical rating scale (NRS) was assessed immediately after the end of the catheterization with 0 being classified as no pain, 1 to 3 being classified as mild pain, 4 to 6 being classified as moderate pain, 7 to 9 being classified as serious pain and 10 being classified as severe pain.

#### 2.3.5. Comfort rating

Changes in patients’ comfort rating were assessed at 1 week and 1 month postoperatively by *Questionnaire on Comfort Rating for PICC Patients*. The questionnaire was developed by Fan Yuying et al with a Cronbach’s α coefficient of 0.687. The scale assessed the patients’ comfort rating after catheterization in terms of psychology, physiology, postoperative arm comfort, and daily living conditions. It consists with a total of 14 items, each item is rated based on 5-grade with the range of 1 to 5, 3 of them were adopted with reverse scoring, the total score ranges from 0 to 70, the lower score indicating greater comfort.

#### 2.3.6. Complications after Catheterization

Counting and comparing the incidence of complications such as dermatitis, seepage, catheter sliding, phlebitis, and blood leakage after catheterization. The judgment criteria of catheter sliding: The length of exposed length of PICC increased more than 1 cm from the time of intubation to the time of extubation. The X-ray film showed that, in its tip position, the upper limb catheterization that located in the superior vena cava or the femoral vein catheterization that located outside the inferior vena cava were regarded as catheter dislodgement, and the exposed length of catheter increased more than 1 cm. The X-ray film showed that, in its tip position, the upper limb catheterization that located in the superior vena cava or the femoral vein catheterization that still located in the inferior vena cava were regarded as catheter sliding.^[15]^ The judgement criteria for phlebitis^[16]^: Grade 0: No symptoms; Grade 1: Rubefaction at the puncture site with or without pain; Grade 2: Pain at the puncture site with rubefaction or edema; Grade 3: Pain at the puncture site with rubefaction, the formation of a cord-like objective, the vein in the cord-like objective can be touched; Grade 4: Pain at the puncture site with rubefaction and pain, the formation of a cord-like objectives, the vein in the cord-like objective can be touched with a length > 1 inch, and pus effusion. Patients with grade 1 or above symptoms can be considered as the onset of phlebitis. The judgment criteria of blood leakage after catheterization: It is normal that a small amount of blood leakage from the dressing within 24 hours, and a blood-soaked area of more than 1 × l cm is considered as blood leakage after catheterization.

### 2.4. Statistical analysis

Data were analyzed by SPSS 23.0 software. Continuous variables such as age, the duration of retention, intraoperative bleeding volume, NRS, comfort rating were tested for normality for confirming that all of them were approximately obeyed a normal distribution and were expressed as mean ± standard deviation, and *t*-tests or Welch’s tests were used to assess significant differences between and within 2 groups at 2 time points, based on the results of the chi-square test. Categorical variables such as gender, catheterization site, catheterization duration, the success rate on PICC puncture, and postoperative complication rate were expressed as percentages, and significant differences between 2 groups were assessed by Pearson chi-square test, Fisher exact test, or Mann–Whitney test. *P* < .05 indicates a statistically significant difference.

## 3. Results

### 3.1. Comparison on baseline characteristics

A total of 200 participants were included in the study. Of these, 100 participants underwent tunnel PICC catheterization and 100 participants underwent B-ultrasound-guided PICC catheterization. Compared with the control group, there was no significant difference in the baseline data characteristics of the observation group, such as age, gender, catheterization site, and the duration of retention, and the difference was not statistically significant (*P* > .05). See Table 1.

**Table 1:** Comparison on baseline characteristics of participants.

Item	Observation group (n = 100)	Control group (n = 100)	*t/X²*	*P*
Age (yr)	50.33 ± 5.82	49.58 ± 5.96	0.636	.526
Gender			0.360	.548
Man	54 (54.00%)	48 (48.00%)		
Woman	46 (46.00%)	52 (52.00%)		
Catheterization site			1.135	.566
Right vena basilica	36 (36.00%)	32 (32.00%)		
Left vena basilica	58 (58.00%)	56 (56.00%)		
Others	6 (6.00%)	12 (12.00%)		
Indwelling time (d)	87.06 ± 11.74	85.70 ± 12.62	0.560	.576

### 3.2. Indwelling time of peripherally inserted central catheter

Compared with the control group, there was no significant difference in the indwelling time of PICC in the observation group, and the difference was not statistically significant (*P* > .05). See Table 2.

**Table 2:** Comparison on indwelling time between 2 groups.

Groups	n	20 min	25 min	30 min
Observation group	100	78 (78.00%)	20 (20.00%)	2 (2.00%)
Control group	100	70 (70.00%)	22 (22.00%)	8 (8.00%)
*Z*		1.027		
*P*		.304		

### 3.3. Success rate on peripherally inserted central catheter puncture

The success rate of PICC puncture in the observation group was higher than that in the control group (*P* < .05). See Table 3.

**Table 3:** Comparison on the success rate of PICC puncture between 2 groups.

Groups	n	One injection	Two injections	Three injections and above
Observation group	100	98 (98.00%)	2 (2.00%)	0 (0.00%)
Control group	100	86 (86.00%)	12 (12.00%)	2 (2.00%)
*Z*		2.199		
*P*		.027		

### 3.4. Intraoperative bleeding volume and numerical rating scale

The intraoperative blood loss in the observation group was 3.23 ± 0.99, the control group was 3.75 ± 1.35. The NRS score of observation group was 2.56 ± 0.98, the control group was 3.01 ± 0.94. Intraoperative bleeding volume and NRS were lower in the observation group than those in the control group (*P* < .05). Please refer Table 4.

**Table 4:** Comparison on intraoperative bleeding volume and NRS between 2 groups.

Groups	n	Intraoperative bleeding volume (g)	NRS
Observation group	100	3.23 ± 0.99	2.56 ± 0.98
Control group	100	3.75 ± 1.35	3.01 ± 0.94
*t*		2.215	2.337
*P*		.029	.021

### 3.5. Comfort rating

At 1 month postoperatively, comfort ratings in the observation and the control groups were lower than those of their same groups at 1 week postoperatively (*P* < .05). At 1 week and 1 month postoperatively, the comfort rating of the observation group were lower than that of the control group (*P* < .05). See Table 5.

**Table 5:** Comparison on the comfort rating of post-catheterization between 2 groups.

Groups	n	1 wk postoperatively	1 mo postoperatively	*t*	*P*
Observation group	100	39.47 ± 5.74	34.01 ± 4.87	5.349	<.001
Control group	100	43.31 ± 7.77	40.33 ± 5.63	2.572	.013
*t*		2.807	6.008		
*P*		.006	<.001		

### 3.6. Postoperative complication rate

In observation group, Dermatitis 4 (4.00%), Catheter sliding 4 (2.00%), Phlebitis 0 (0.00%), Post-intubation blood leakage (within 24 hours) 4 (2.00%). However in the control group, Dermatitis 2 (2.00%), Catheter sliding 6 (6.00%), Phlebitis 8 (8.00%), Post-intubation blood leakage (within 24 hours) 8 (8.00%). The postoperative complication rate in the observation group was lower than that in the control group (*P* < .05). See Table 6.

**Table 6:** Comparison of postoperative complication rates between 2 groups.

Item	Observation group (n = 100)	Control group (n = 100)	*X²*	*P*
Dermatitis	4 (4.00%)	2 (2.00%)		
Catheter sliding	4 (2.00%)	6 (6.00%)		
Phlebitis	0 (0.00%)	8 (8.00%)		
Post-intubation blood leakage (within 24 h)	4 (2.00%)	8 (8.00%)		
Total incidence	8 (8.00%)	24 (24.00%)	4.761	.029

## 4. Discussion

The results of this study showed that the success rate of initial catheterization in the observation group was significantly better than that in the control group. TPICC adds a section of subcutaneous tunnel between catheter exit and puncture site. TPICC selects a suitable exit point (at least 2 cm from the puncture site) in the middle third of the upper arm, uses a tunnel needle to puncture from the exit site to the puncture site of the PICC, connects the PICC to the tunnel needle, and leads it out to the exit point. This subcutaneous tunnel expands the range of choices for the puncture point and the exit. On the one hand, it offers a therapeutic means for patients who cannot undergo conventional PICC; On the other hand, on the basis of conventional PICC, the puncture point of tunnel PICC can be selected from veins with larger diameter, which increases the success rate of catheterization. In clinical practice, some patients, such as pediatric patients, the patients with long-term malnutrition and marasmus plus intravenous infusion, or the patients with localized phlebitis. Their veins have small diameters. It is difficult to find ideal peripheral veins for puncture. Subcutaneous tunnels increase the selection range of puncture points and exits, provide more opportunities for puncture among patients who are not able to undergo conventional PICC, and expand the applying scope of PICC.^[17]^ At the same time, combining with the Seldinger puncture technique, the puncture point of tunnel PICC can be chosen from veins with larger diameters, such as the veins nearing the proximal side, which is also conducive to the increase on the success rate of catheterization.^[18]^ The incidence of mechanical phlebitis is also reduced when the success rate of initial catheterization is high.^[19]^ When creating a subcutaneous tunnel, the vessels and needle eye are effectively staggered so that the catheter is less likely to slide in and out of the puncture point, which is consistent with the results of the study.^[20,21]^

In addition, ultrasound-assisted Seldinger puncture is commonly used for routine puncture; however, it is associated with numerous complications.^[22]^ When complications arise during PICC catheterization, it significantly adds to the economic, psychological, and physical burden on patients. Clinical practice has demonstrated that preventing PICC complications is more effective than treating them.^[23–25]^ Therefore, it is imperative to enhance the puncture method to improve work efficiency and minimize complications. The most common complication after PICC is mechanical phlebitis, which is an acute aseptic inflammation that occurs when the vein wall is damaged by various causes.^[26]^ Bacteria entering the bloodstream along the catheter from the catheterization site of skin, which is the main cause of catheter-related infections. As an open channel existing between the blood vessel and the outside world, it can result in localized infections or even systemic infections. The results of this study showed that no phlebitis occurred in the observation group, while there were 8 cases of phlebitis in the control group, and the comfort rating of the observation group were lower than that of the control group at 1 week and 1 month postoperatively (*P* < .05). The incidence of phlebitis in 8 patients from the control group may be related to the successful puncture with more than 2 needles, the poor condition of vessel, which caused damage to the vessel wall and the long time of catheterization. This study suggests that PICC catheterization can effectively prevent certain complications. The reasons for this are analyzed as follows: TPICC catheterization reduces mechanical friction between the catheter and the vascular wall, thereby decreasing the occurrence of mechanical phlebitis.^[27,28]^ TPICC catheterization can compress blood vessels when skin tissue contracts,^[29]^ effectively reducing the risk of catheter shedding. TPICC catheterization is a relatively new and low-risk method of catheterization.^[30]^ In the traditional PICC mode of femoral vein puncture in the middle part of the thigh, the puncture point is located on the blood vessel, which can cause mechanical damage to the skin and blood vessels. After withdrawing the puncture needle, blood can ooze from the puncture point.^[31]^ However, TPICC catheterization creates a subcutaneous tunnel that effectively separates the catheter outlet from the vascular puncture site, maintaining a safe distance. Additionally, the skin has the ability to self-contract, which further compresses the vascular puncture site. Due to these advantages, tunnel catheterization can control bleeding and reduce the bleeding rate.^[32]^ Tunnel PICC insertion involves creating a subcutaneous tunnel between the puncture location and the catheter outlet site. This expands the options for exit location and puncture point, allowing for the selection of a vein with a larger diameter at the tunnel PICC insertion point. This increases the success rate of catheter insertion, especially in patients with small vein diameters where finding an ideal peripheral vein during puncture can be challenging. TPICC can prevent bacteria from migrating from the skin to the deep vein, which can reduce the incidence of catheter-related infections.^[19]^ Moreover, the lower incidence of mechanical phlebitis with TPICC may also be related to the reduction of mechanical friction between the catheter and the vessel wall.^[33]^ This improves the quality of PICC, maximizes the benefits of PICC, and improves patients’ comfort.

The tissue contraction from skin can play a fixation in the catheter. This reduced the incidence of catheter sliding after catheterization due to patient activities and other reasons often lead to the catheter sliding in the puncture point needle eye, the tissue contraction from skin could play a fixation in the catheter so that the catheter cannot be easily sliding out^[34]^; As the polyester fiber sleeve generally takes 3 to 4 weeks to be fixed and wrapped by the connective tissue. TPICC makes the exit at skin and the puncture point needle eye at vessel staggered, a distance apart, increasing the friction of catheter sliding, so that the tube is not easy to slide inside and outside the puncture point. One case of catheter sliding occurred in the observation group. The patient’s malignant disease, loose skin, loose dressing, and lack of timely maintenance led to catheter sliding with 2 cm, and other 3 cases of catheter sliding occurred in the control group, which may be related to the patient’s loose skin or poor compliance.

Blood seepage from the puncture site is one of the common complications of PICC, and most of them occur within 24 hours after puncture, which is often caused by elbow extension and flexion, upper limb support exertion, and so on. As a new method of catheterization, its tunnel needle used in tunneling is a mature product in the market, and the risk associated with the procedure is low. Compared with the conventional method, the tunnel PICC technique has more advantages in preventing complications such as blood seepage, mechanical phlebitis, and secondary displacement after catheter dislodgement, and it can effectively improve the effect of catheterization. The puncture point of conventional PICC is above the vessel, the vessel and the skin puncture point are in the same position, the needle blade can cause the mechanical-cutting damage to the blood vessel and the skin, and blood can leak out around the puncture point of catheterization after the needle is withdrawn.^[26]^ TPICC requires the creation of subcutaneous tunnels, so that the vascular puncture needle eye and catheter exit are effectively staggered, through which a safe distance can be formed, and when the skin tissue shrinks, it can also play a role in compressing the puncture needle eye of vessel. Thanks to the advantages of TPICC, the needle eye is compressed, so that the patient’s blood leakage can be controlled, thus effectively reducing the incidence of blood leakage. The results of this study showed that there were l cases of blood leakage within 24 hours in the observation group, which might be related to the patients’ platelets being lower than normal values. In the control group, there were 4 cases of blood leakage in 24 hours, which might be related to the early activities of the patients.

However there are limitations to this study. For example, the small sample size included and the failure to observe changes in comfort after catheterization over a longer period of follow-up. These may lead to some bias in the results of the study. Future studies need to expand the sample size and improve the study design for further investigating the results of current study.

In summary, the application of TPICC technology improves the success rate of puncture and leads the catheter to the optimal location for exiting. Avoiding the lesion site with scar, there is a certain distance between the vascular puncture point and the skin on catheter exit, which plays the role of a natural barrier and this can reduce the probability of blood flow-related infections and phlebitis. For patients with poor coagulation or hypoproteinemia, alleviating their local blood leakage. For patients with skin laxity, due to subcutaneous tissues can fix the catheter, thus reducing the rate of catheter sliding, facilitating the daily maintenance, and improving the patients’ comfort rating. It improves the quality of life among patients to a great extent and achieves the purpose on the double safety of infusion.

## Authors contributions

**Conceptualization:** Yingshan Zheng, Xia Xiang, Li Zhang, Shaoli He.

**Data curation:** Yingshan Zheng, Lixiang Li, Li Zhang.

**Formal analysis:** Yingshan Zheng, Xia Xiang, Lixiang Li, Li Zhang, Shaoli He.

**Funding acquisition:** Yingshan Zheng, Xia Xiang, Lixiang Li, Li Zhang.

**Investigation:** Yingshan Zheng, Xia Xiang.

**Methodology:** Xia Xiang, Lixiang Li, Shaoli He.

**Visualization:** Li Zhang, Shaoli He.

**Writing – original draft:** Yingshan Zheng, Xia Xiang, Lixiang Li, Li Zhang, Shaoli He.

**Writing – review & editing:** Yingshan Zheng, Xia Xiang, Lixiang Li, Li Zhang, Shaoli He.
